# BMP Signaling in the Human Fetal Ovary is Developmentally Regulated and Promotes Primordial Germ Cell Apoptosis

**DOI:** 10.1002/stem.440

**Published:** 2010-05-06

**Authors:** Andrew J Childs, Hazel L Kinnell, Craig S Collins, Kirsten Hogg, Rosemary AL Bayne, Samira J Green, Alan S McNeilly, Richard A Anderson

**Affiliations:** a Medical Research Council Human Reproductive Sciences Unit and Developmental Sciences, Centre for Reproductive Biology, Queen's Medical Research InstituteEdinburgh, United Kingdom; a University of Edinburgh Division of Reproductive and Developmental Sciences, Centre for Reproductive Biology, Queen's Medical Research InstituteEdinburgh, United Kingdom

**Keywords:** Bone morphogenetic protein, SMAD, Primordial germ cell, Ovary, Oocyte, Apoptosis

## Abstract

Primordial germ cells (PGCs) are the embryonic precursors of gametes in the adult organism, and their development, differentiation, and survival are regulated by a combination of growth factors collectively known as the germ cell niche. Although many candidate niche components have been identified through studies on mouse PGCs, the growth factor composition of the human PGC niche has not been studied extensively. Here we report a detailed analysis of the expression of components of the bone morphogenetic protein (BMP) signaling apparatus in the human fetal ovary, from postmigratory PGC proliferation to the onset of primordial follicle formation. We find developmentally regulated and reciprocal patterns of expression of *BMP2* and *BMP4* and identify germ cells to be the exclusive targets of ovarian BMP signaling. By establishing long-term cultures of human fetal ovaries in which PGCs are retained within their physiological niche, we find that BMP4 negatively regulates postmigratory PGC numbers in the human fetal ovary by promoting PGC apoptosis. Finally, we report expression of both muscle segment homeobox *(MSX)1* and *MSX2* in the human fetal ovary and reveal a selective upregulation of *MSX2* expression in human fetal ovary in response to BMP4, suggesting this gene may act as a downstream effector of BMP-induced apoptosis in the ovary, as in other systems. These data reveal for the first time growth factor regulation of human PGC development in a physiologically relevant context and have significant implications for the development of cultures systems for the in vitro maturation of germ cells, and their derivation from pluripotent stem cells.

## INTRODUCTION

Primordial germ cells (PGCs) are a transient population of germline stem cells present only during embryonic development and are the precursors of sperm and egg in the adult organism [1]. PGCs arise from the proximal epiblast of the mammalian embryo [2,3] before migrating to and colonizing the developing gonadal ridges, during which their numbers increase substantially by mitotic proliferation. Following sex determination, PGCs in the nascent ovary that differentiate into oogonia; a population of transit-amplifying cells which are irreversibly committed to meiosis, but which first undergo several rounds of mitotic proliferation yielding syncitial cysts or “nests” of germ cells [4]. Subsequent breakdown of these nests releases oocytes to associate with surrounding somatic cells to form primordial follicles [5,6]. However, the spatiotemporal organization of these processes differs considerably between rodents and humans. In the mouse, postmigratory PGC proliferation, meiotic entry and arrest, and follicle formation occur in three broadly synchronized waves over a period of around 10–12 days (embryonic day [E] 10.5 to postnatal day [P] 2) [7]. In contrast, the same events in the human occur over a period of months and overlap considerably, such that germ cells at all stages of development are present in the fetal ovary at later gestations [8–10].

In common with stem cells in other systems, PGCs have the capacity to undergo self-renewal (proliferation), differentiation (entry into meiosis), or programmed cell death (apoptosis). Achieving an appropriate balance of these cell-fate decisions is critical, as the oocyte population formed and assembled into primordial follicles in utero provides the basis for the future reproductive lifespan of the adult female (although some aspects of this have recently been questioned [11,12]). PGC behavior is therefore tightly regulated by the surrounding gonadal microenvironment; a combination of growth factors, contacts with neighboring somatic cells, and interactions with the extracellular matrix that collectively make up the germ cell niche. Although genetic approaches have revealed a small number of growth factors, such as bone morphogenetic protein seven (BMP7), stromal derived factor one (SDF-1), and stem cell factor (SCF) to be essential components of the PGC niche [13–15], much of our understanding of its growth factor composition has been derived from in vitro studies on isolated PGCs cultured on feeder cells (reviewed in [16]). These findings have not always supported results in vivo, however, suggesting feeders may not accurately recapitulate the germ cell niche in vivo [17–20].

Members of the BMP subgroup of the transforming growth factor-β (TGF-β) superfamily of growth factors play essential roles throughout mammalian gametogenesis. In mice, the formation and proliferation of the PGC precursor population is dependent on Bmps 2, 4, and 8b [21–24], whereas Bmp4 has also been shown to increase migratory PGC numbers in embryo slice cultures [25]. The role of BMP signaling in regulating the behavior of postmigratory PGC development is less clear: culture of fetal mouse ovaries with Bmp2 or Bmp4 reduced the number of meiotic germ cells [26], yet Bmp4 has also been reported to promote the proliferation of isolated postmigratory PGCs cultured on feeder layers in vitro [27]. To date, no roles for BMPs in regulating the behavior of meiotic germ cells or in the regulation of human germ cell development have been ascribed.

BMPs regulate a range of developmental processes including proliferation, differentiation, and apoptosis in a tissue- and developmental-stage specific fashion [28]. BMPs promote apoptosis in a diverse range of developmental settings, including embryo cavitation [29], brain and eye development [30–32], and digit morphogenesis [33,34]. In many of these instances expression of the muscle segment homeobox (*MSX*) genes correlates strongly with the location of BMP-induced apoptosis [30,31,35] suggesting these factors to be downstream effectors of BMP-induced cell death. Consistent with this, MSX2 can itself promote apoptosis in tissues known to undergo cell death in response to BMP signaling [36,37] and knockdown of *MSX2* expression ablates the proapoptotic effects of BMPs [38,39]. Although proposed [40], a proapoptotic function for BMP signaling during early germ cell development has not been extensively studied, and to date, no clear data exist regarding the expression of *MSX1* and *MSX2* in the developing mammalian ovary.

Here, we report the existence and characterization of a developmentally regulated BMP-signaling system within the human fetal ovary and demonstrate a proapoptotic effect of BMP treatment on human PGCs in long-term cultures of human fetal ovaries, demonstrating for the first time regulation of human PGC fate by growth factor signaling in a physiologically representative system.

## MATERIALS AND METHODS

### Tissue

Morphologically normal first- and second-trimester ovaries (8–20 weeks gestation) were obtained after medical termination of pregnancy. Maternal consent was obtained, and the study was approved by the Lothian Research Ethics Committee. Gestation was determined by ultrasound scan and (for second trimester specimens) confirmed by subsequent direct measurement of foot length. Sex of first trimester specimens was determined by PCR genotyping for the *SRY* gene [41]. Ovaries were dissected into sterile Hank's Balanced Salt Solution (HBSS; Invitrogen, Paisley, U.K.) before being snap-frozen and stored at –80°C (for RNA extraction), fixed in Bouin's solution and processed into paraffin using standard methods (for immunohistochemical analysis), or cultured as detailed below.

### RNA Extraction and cDNA Synthesis

Total RNA was extracted from human fetal ovaries using the RNeasy Micro Kit or RNeasy Mini Kit (QIAGEN, Crawley, U.K.) with on-column DNaseI digestion according to the manufacturer's instructions. For determination of gene expression across gestation, gonads were dissected free of mesonephric tissue before RNA extraction. First strand cDNA was synthesized using the Superscript III Reverse Transcriptase Master Mix (Invitrogen) as per the manufacturer's instructions. Duplicate reactions in which the Reverse Transcriptase enzyme was omitted were set up as negative controls.

### qRT-PCR Analysis

Quantitative RT-PCR was performed as described previously [42]. Primer sequences are detailed in Table 1. Standard curves for each PCR amplicon were generated by plotting Ct values from cDNA dilutions (1:5–1:10,000) of human fetal ovary cDNA, against log concentration, and the resulting slope used to calculate gene expression in experimental samples. To permit comparison between samples, expression of each amplicon was calculated relative to expression of the housekeeping gene *RPL32*.

**Table 1:** Oligonucleotide primer sequences used in qRT-PCR

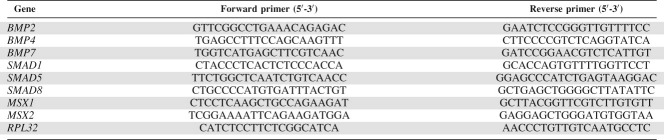

### Immunofluorescence and Immunohistochemistry

Immunofluorescence was performed as described previously [43], using rabbit polyclonal antibodies raised against BMPR1a, BMPR1b (gifts of Dr C. Helldin, Karolinska Institute, Sweden, both diluted 1:1,000), and pSMAD1/5/8 (New England Biolabs, Hitchin, U.K.; #9511, diluted 1:1,000 [first trimester specimens] and 1:500 [second trimester specimens)). Following PBS washes, slides were incubated with goat anti-rabbit peroxidase secondary antibody (Vector Laboratories, Peterborough, U.K.; 1:200 dilution), then incubated in TSA Plus Fluorescein Green (Perkin Elmer, MA, USA; diluted 1:50) for 2 minutes at room temperature. Slides were counterstained with propidium iodide and mounted using PermaFluor Mounting Medium (Beckman Coulter, High Wycombe, U.K.). Fluorescent images were captured using a LSM510 confocal microscope (Carl Zeiss, Welwyn Garden City, U.K.).

Immunohistochemistry was performed as previously described [44]. Slides were incubated in primary antibody (rabbit polyclonal antibodies to activator protein-2 gamma [AP-2γ; Santa Cruz Biotechnology Inc., Santa Cruz, CA, http://www.scbt.com; #sc-8977], SMAD6 [Imgenex, CA, USA; #IMG-555] and cleaved caspase three (New England Biolabs; #9601) [all diluted 1:100 in tris buffered saline [TBS] supplemented with 20% normal goat serum [NGS] and 5% BSA, except SMAD6, diluted 1:50]) at 4°C overnight. Primary antibodies were detected using a biotinylated goat anti-rabbit secondary antibody (Dako, Cambridge, U.K.), diluted 1:500 in TBS/NGS/BSA, and incubated for 1 hour at room temperature. Staining was visualized using streptavidin-horseradish peroxidase (diluted 1:1,000 in TBS) followed by 3,3′-diaminobenzidine tetrahydrochloride (DAB; Dako). Immunohistochemical detection of phosphorylated histone H3 (phospho-H3) was performed on an automated Bond Immunostaining Robot using a rabbit polyclonal to phospho-H3 (Upstate Biotechnology, Milton Keynes, U.K., #06-570) as the primary antibody. Images were captured using an Olympus Provis microscope (Olympus, London, U.K.).

### Culture of Fetal Ovaries

Human fetal ovary-mesonephros complexes (58–65 days gestational age) were cultured on cell culture inserts (Greiner Bio-One, Stonehouse, U.K.) in serum free medium (αMEM + GlutaMAX with 1× nonessential amino acids [Invitrogen]; 2 mM sodium pyruvate and 3 mg/ml bovine serum albumin (BSA) Fraction V [both from Sigma, Poole, U.K.]; and penicillin/streptomycin/amphotericin B [Cambrex Biosciences, MD, USA]) in the presence or absence of 100 ng/ml recombinant human BMP4 (rhBMP4; Invitrogen) for 24 hours (to determine effects on *MSX* gene expression) or 10 days (to determine effects on PGC number, proliferation and apoptosis) in a humidified incubator (37°C, 5% CO_2_). In 10 days cultures, a complete medium change was performed every 48 hours. After culture, tissues were either lysed in RLT buffer for subsequent RNA extraction, or fixed and processed into paraffin for histological assessment as detailed above.

### Stereological Determination of Germ Cell number, Proliferation, and Apoptosis

Immunohistochemistry to detect AP-2γ, phospho-H3, and cleaved caspase three was performed on adjacent serial sections as outlined above. Total, apoptotic and proliferating PGC numbers were determined using a Zeiss Axio Imager A1 microscope (Carl Zeiss) fitted with a camera and automatic stage (Prior Scientific Instruments Ltd., Cambridge, U.K.). Image Pro Plus software 4.5.1 with Stereologer Pro five software (Media Cybernetics, Workingham, U.K.) was used for cell counts and determination of areas. Total, proliferating and apoptotic PGC numbers were counted using the point-counting tool, and total numbers per ovary calculated according to the method detailed in [45]. Numbers of AP-2γ-positive cells were determined using every fifth section, whereas phospho-H3 and cleaved caspase three counts were determined using every 10th section.

### Statistical Analysis

RT-PCR data were analyzed by ANOVA (gestational changes) or paired *t* tests (experimental manipulations) using GraphPad Prism 5.0 software (GraphPad Software, CA, USA).

## RESULTS

### Expression of BMP Ligands Is Developmentally Regulated in the Human Fetal Ovary

**Figure 1:**
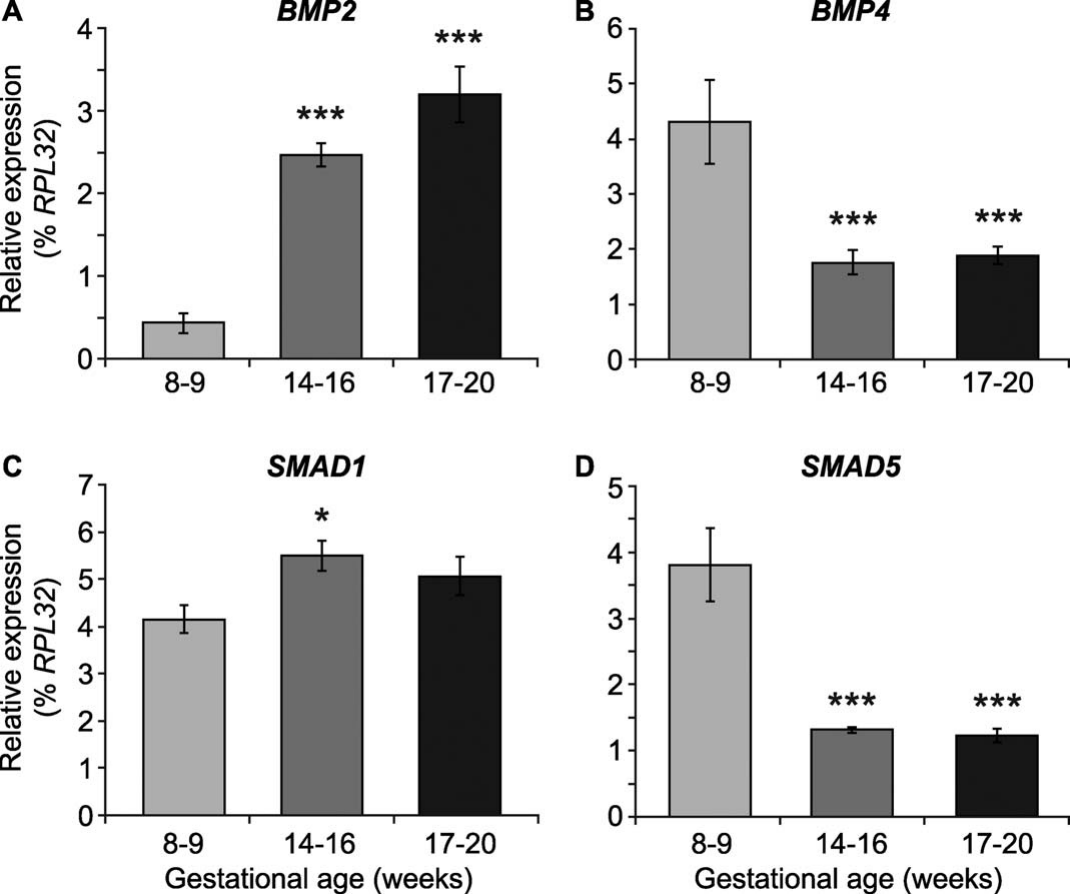
Expression of genes encoding BMP ligands and SMADs is developmentally regulated during human fetal ovarian development. Expression of genes encoding BMP ligands and downstream SMAD proteins in the human fetal ovary was analyzed by qRT-PCR across a gestational range, from postmigratory primordial germ cell (PGC) proliferation (8–9 weeks gestation), through the formation of syncitial clusters of germ cells and onset of meiosis (14–16 weeks) to the formation of the first primordial follicles (17–20 weeks). (A) Expression of *BMP2* increased significantly between 8–9 and 14–16/17–20 weeks gestation, concomitant with the onset of germ cell differentiation. (B) *BMP4* expression decreased markedly over the same period (***p < .001, *n* = 5–6). (C) Expression of *SMAD1* increased slightly between 8–9 and 14–16 weeks (*p < .05, *n* = 5–6), but was not significantly higher at 17–20 weeks than 8–9 weeks. (D) In contrast, *SMAD5* expression declined sharply from 8–9 to 14–16 weeks (***p < .001, *n* = 5–6), mirroring the fall in *BMP4* expression. Abbreviations: BMP2, Bone morphogenetic protein two; BMP4, Bone morphogenetic protein four.

We first investigated the expression profiles of *BMP2*, *BMP4*, and *BMP7*. We performed quantitative RT-PCR on specimens grouped by gestational age to broadly represent three key developmental stages in human fetal ovarian germ cell development; 8–9 weeks gestation (mitotic PGC proliferation only), 14–16 weeks gestation (formation of syncitial clusters of oogonia and onset of meiotic germ cell differentiation), and 17–20 weeks gestation (breakdown of syncitial clusters and assembly of primordial follicles), although considerable overlap exists at later gestations. *BMP2* expression increased significantly with gestation, rising from 0.43% ± 0.12% of expression of the housekeeping gene *RPL32* at 8–9 weeks gestation to 3.2% ± 0.34% at 17–20 weeks (*n* = 5–6, *p* < .0001, Fig. 1A), a ∼7.4-fold increase. The majority of this increase occurred between 8–9 and 14–16 weeks, with a ∼5.7-fold increase (*p* < .0001). In contrast, we detected a significant decrease in the expression of transcripts encoding BMP4 with increasing gestation. *BMP4* gene expression was highest at 8–9 weeks, and declined sharply at 14–16weeks (4.3% ± 0.8% at 8–9 weeks, vs. 1.8% ± 0.2% at 14–16weeks, *n* = 5–6, *p* < .001; Fig. 1B) and remained low at 17–20 weeks (1.9% ± 0.2%). It appears therefore that reciprocal changes occur in the expression of *BMP2* and *BMP4* concomitant with the onset of germ cell differentiation.

We also examined the expression of *BMP7. BMP7* transcript levels were very low, ∼9,000 times lower than those encoding *BMP4. BMP7* expression decreased approximately fivefold over the period examined (4.66 × 10^−5^ % to 1.09 × 10^−5^%, *n* = 5–6) but this was not statistically significant (not shown). These data suggest that BMP7 may not play a significant role in human fetal germ cell development in contrast to the situation in mice [13].

### Changes in Expression of Intracellular SMADs Parallel Changes in BMP Expression

BMP receptor-regulated (BR-)SMADs 1, 5, and 8 transduce BMP signals from the membrane to the nucleus. We detected a small but significant increase in the expression of transcripts encoding SMAD1 between 8–9 and 14–16 weeks (4.14% ± 0.30% to 5.50% ± 0.32%, *p* < .05; Fig. 1C), although the levels did not differ significantly between 8–9 and 17–20weeks (4.14% ± 0.30% vs. 5.06% ± 0.40%). In contrast, we detected a sharp decline in the expression of *SMAD5*, which paralleled the fall in *BMP4* expression. *SMAD5* expression was significantly higher at 8–9 weeks than at 14–16 or 17–20 weeks (3.8% ± 0.6% at 8–9 weeks, vs. 1.3% ± 0.05% at 14–16 weeks and 1.2% ± 0.1% at 17–20 weeks, *n* = 5–6, *p* < .001; Fig. 1D). We were unable to detect *SMAD8* expression at any gestational stage examined. Together, these data suggest a switch in the predominantly expressed SMAD may occur coincident with the onset of germ cell differentiation in the human fetal ovary.

### Germ Cells Are the Targets of BMP Signaling in the Human Fetal Ovary

**Figure 2:**
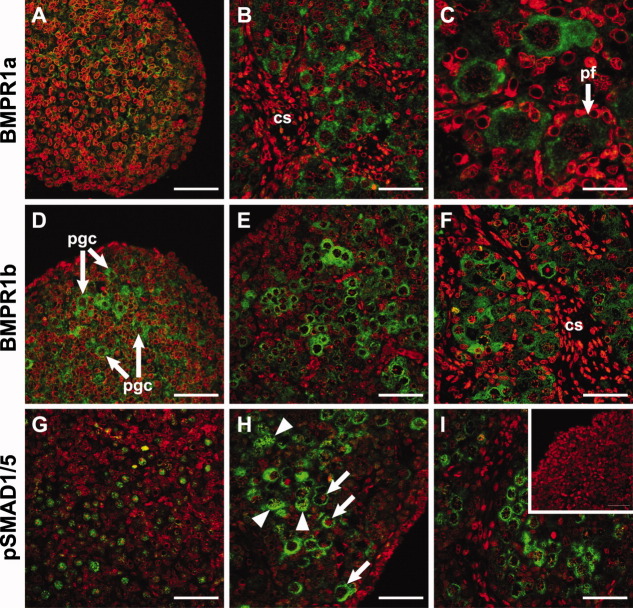
Germ cells express BMP receptors and are the exclusive targets of ovarian BMP signaling. Immunohistochemical localization of type I BMP receptors BMPR1a (**A–C**) and BMPR1b (**D–F**) in the human fetal ovary. Expression of BMPR1a in the first trimester (9 weeks gestation) human fetal ovary is diffuse, with expression detectable in both germ and somatic compartments (**A**), but expression becomes restricted to germ cells at later gestations (**B, C**: 19 weeks), with no expression detectable in stromal cell streams. Following the breakdown of syncitial clusters of germ cells, single oocytes and those in primordial follicles continue to express BMPR1a (19 weeks, **C**). In contrast, BMPR1b expression is germ cell specific at all gestations examined, with intense expression detectable in PGC at 9 weeks (**D**) and in clusters of germ cells at 14 (**E**) and 19 weeks (**F**) gestation. (**G–I**): Immunohistochemical localization of phosphorylated (p)SMAD1/5. At 9 weeks gestation, pSMAD1/5 is detectable only in the nuclei of primordial germ cells. At 19 weeks (**H**), a mixture of germ cells displaying solely cytoplasmic (arrows) and cytoplasmic and nuclear (arrowheads) staining can be detected in close proximity, whereas other clusters appear to contain germ cells with exclusively cytoplasmic staining (**I**). (Inset in **I**: negative control omitting primary antibody). Green: (**A–C**) BMPR1a; (**D–F**) BMPR1b; (**G–I**) pSMAD1/5; red: propidium iodide. Scale bars: (**A, B, D–I**) 50 μm, (**C**) 125 μm. Abbreviations: BMPR, BMP receptor; CSs, cell streams; PFs, primordial follicles; PGCs, primordial germ cells; pSMAD, phosphoSMAD.

We performed fluorescent immunohistochemistry to identify the specific cell types expressing the BMP receptors BMPR1a (ALK3) and BMPR1b (ALK6). At 9 weeks gestation, we detected weak diffuse staining for BMPR1a throughout the gonad, in both germ and somatic cells (Fig. 2A). In contrast, BMPR1b expression was clearly confined to PGCs (Fig. 2D). By 14 weeks, staining for both receptors was restricted to germ cells, a pattern which persisted to 19 weeks gestation (Fig. 2B, 2C, 2E, 2F). BMPR1a and BMPR1b were detectable both in germ cells in syncitial clusters (Fig. 2B, 2E, 2F) and at later gestations in single oocytes in the process of associating with surrounding somatic cells to form primordial follicles (Fig. 2C). We therefore conclude that expression of these proteins is restricted to germ cells, which are thus the target of BMP signaling in the human fetal ovary.

### The Subcellular Localization of Phosphorylated SMAD1/5 Changes on Germ Cell Differentiation

To determine whether germ cells are actively receiving and transducing BMP signals, we performed immunohistochemistry to detect the active, phosphorylated (p-) isoforms of SMAD1 and SMAD5. pSMAD1/5 staining was restricted to germ cells at all gestations (9, 14, and 19 weeks) examined (Fig. 2G–2I). At 9 weeks, pSMAD1/5 staining was detected exclusively in PGC nuclei (Fig. 2G), consistent with a previous report identifying these cells to be the targets of BMP action in the mouse gonad at a comparable developmental stage [46]. At 14 and 19 weeks gestation, germ cells continued to stain strongly and exclusively for pSMAD1/5, but the predominant subcellular localization of pSMAD1/5 changed dramatically, with almost all pSMAD1/5 staining localizing to the germ cell cytoplasm (Fig. 2H, 2I). In some germ cells, pSMAD1/5 appeared to localize exclusively to the cytoplasm, whereas in others staining was detectable in both the nucleus and cytoplasm (Fig. 2H). Germ cells with exclusively nuclear staining were rare.

**Figure 3:**
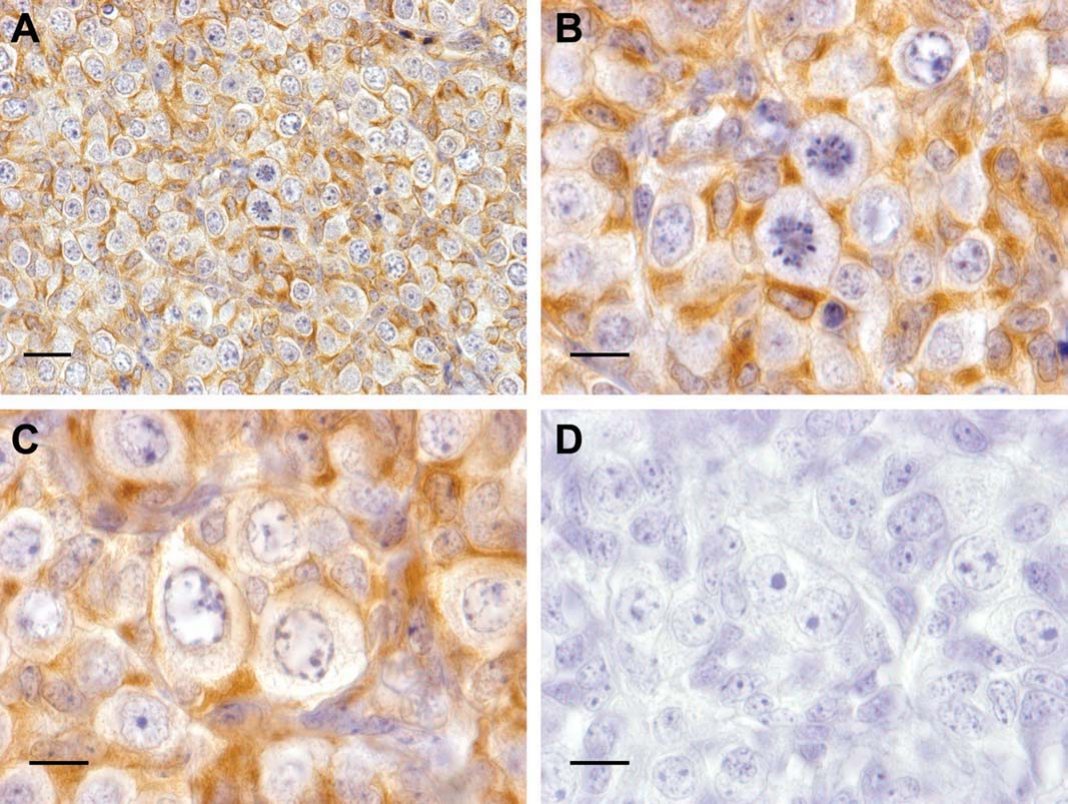
SMAD6 is expressed only in the somatic compartment of the human fetal ovary. Representative photomicrographs of a section of human fetal ovary stained for SMAD6. (**A**): At low magnification, large numbers of immunonegative germ cells can be seen surrounded by a meshwork of immunopositive somatic cells (brown staining). (**B, C**): High magnification photomicrographs showing immunopositive somatic cells (brown staining) surrounding immunonegative germ cells of different sizes and developmental stages. (**D**): Negative control omitting primary antibody. Scale bars: (**A**) 20 μm, (**B–D**) 10 μm.

Transduction of BMP signals from the cell membrane to the nucleus requires the association of pSMAD1/5/8 with the common mediator SMAD4, which facilitates import of the “activated” SMAD complex into the nucleus. This process can be antagonized by the action of an Inhibitory (I-)SMAD, SMAD6, which competes with SMAD4 for binding of pSMAD1 [47]. To determine whether SMAD6 was responsible for the cytoplasmic localization of pSMAD1/5 in differentiating germ cells, we performed immunohistochemstry for SMAD6 on sections of 14 week gestation human fetal ovary (Fig. 3A–3C). Strikingly, we found SMAD6 to localize exclusively to the cytoplasm of ovarian somatic cells interspersed within and around clusters of immunonegative oogonia. SMAD6 therefore does not attenuate BMP signaling in human fetal germ cells by restricting nuclear translocation of pSMAD1/5, but may insulate the somatic compartment against BMP signals.

### BMP4 Negatively Regulates PGC Numbers in Long-Term Cultures of Human Fetal Ovaries

**Figure 4:**
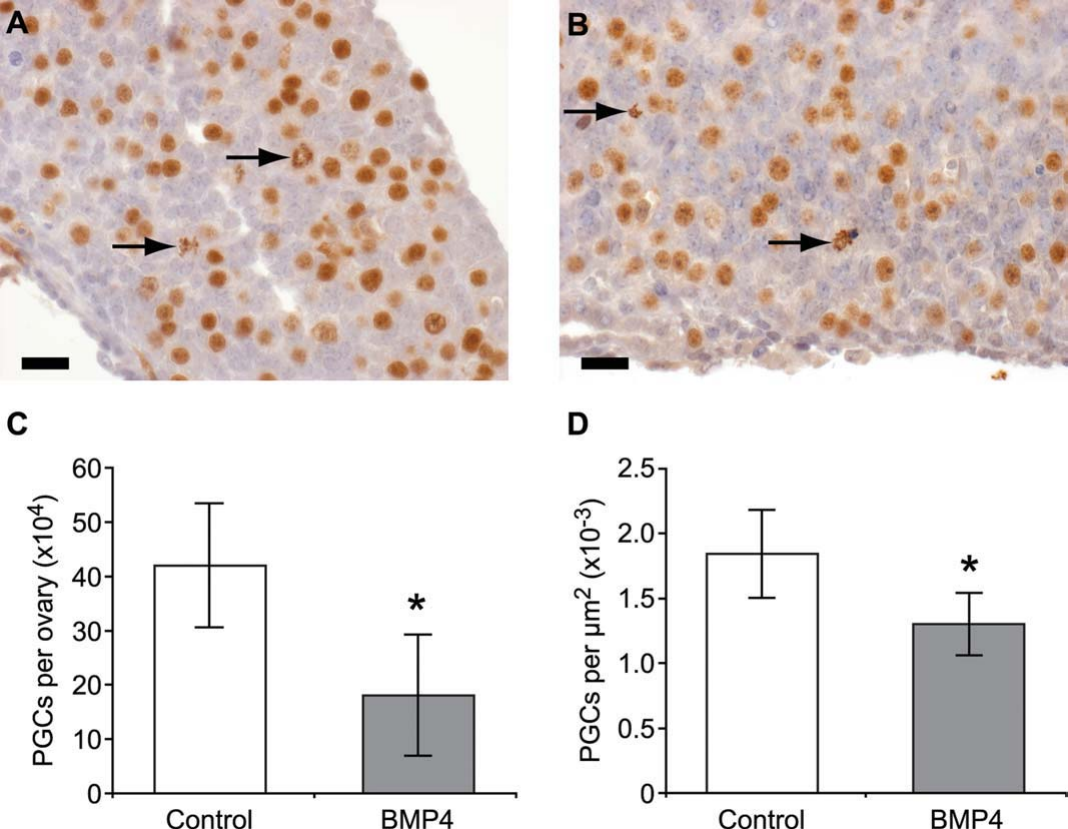
BMP4 negatively regulates PGC numbers in human fetal ovaries cultured in vitro. Representative photomicrographs of sections of human fetal ovaries maintained in serum-free culture for 10 days, stained for the germ cell marker activator protein-2gamma (AP-2γ), showing abundant PGCs (brown staining) in both control (**A**) and BMP4-treated (**B**) cultures. Mitotic PGCs are also detectable (arrows). Stereological determination of AP-2γ-positive PGC numbers reveals a significant decrease in the number of PGCs per ovary in BMP-treated cultures compared with controls (**C**; *p* < .04, *n* = 4), and in the number of PGCs per square micrometer (**D**; *p* < .04, *n* = 4), revealing negative regulation of PGC numbers by BMPs in the human fetal ovary. Scale bars: (**A, B**) 10 μm. Abbreviations: BMP4, bone morphogenetic protein four; PGCs, primordial germ cells.

To determine the role of BMP4 in regulating human PGC development, we cultured gonad-mesonephros complexes with or without human recombinant Bone Morphogenetic Protein 4 (hrBMP4). Immunohistochemistry for activator protein two gamma (AP-2γ), a transcription factor expressed specifically by human PGCs and pluripotent germ cell tumor cells [48] revealed germ cells to be abundant in control and hrBMP4-treated ovaries, and highlighted the presence of actively proliferating germ cells within the explanted gonads (Fig. 4A, 4B). Our culture conditions therefore support both proliferation and survival of human PGCs in the absence of exogenous growth factors.

We then performed stereological assessment of serial sections to determine total germ cell number and density. Control ovaries contained an average of 42,002 ± 11,455 PGCs, a figure consistent with the ∼50,000 germ cells reported to be present in the human fetal ovary at a comparable developmental stage in vivo [49] and confirming that germ cells survive well in our culture system. The addition of hrBMP4 for 10 days had a dramatic effect on PGC number, causing a significant reduction to 18,095 ± 11,116 PGCs (*n* = 4, *p* = .03); a decrease in germ cell number of 57% compared with untreated controls (Fig. 4C). Total ovarian area was reduced slightly in the hrBMP4-treated gonads compared with untreated controls, but this did not reach statistical significance (not shown). To correct for this, we calculated the germ cell density, that is, the number of PGCs per unit area. PGC density was approximately 30% lower in hrBMP4-treated gonads compared with untreated controls (1.30 ± 0.23 × 10^−3^ vs. 1.84 ± 0.32 × 10^−3^ cells per square micrometer, *n* = 4, *p* = .03; Fig. 4D). These data reveal that BMP4 negatively regulates PGC numbers in the first trimester human fetal ovary.

### BMP4 Promotes PGC Apoptosis, But Does Not Impair PGC Proliferation

**Figure 5:**
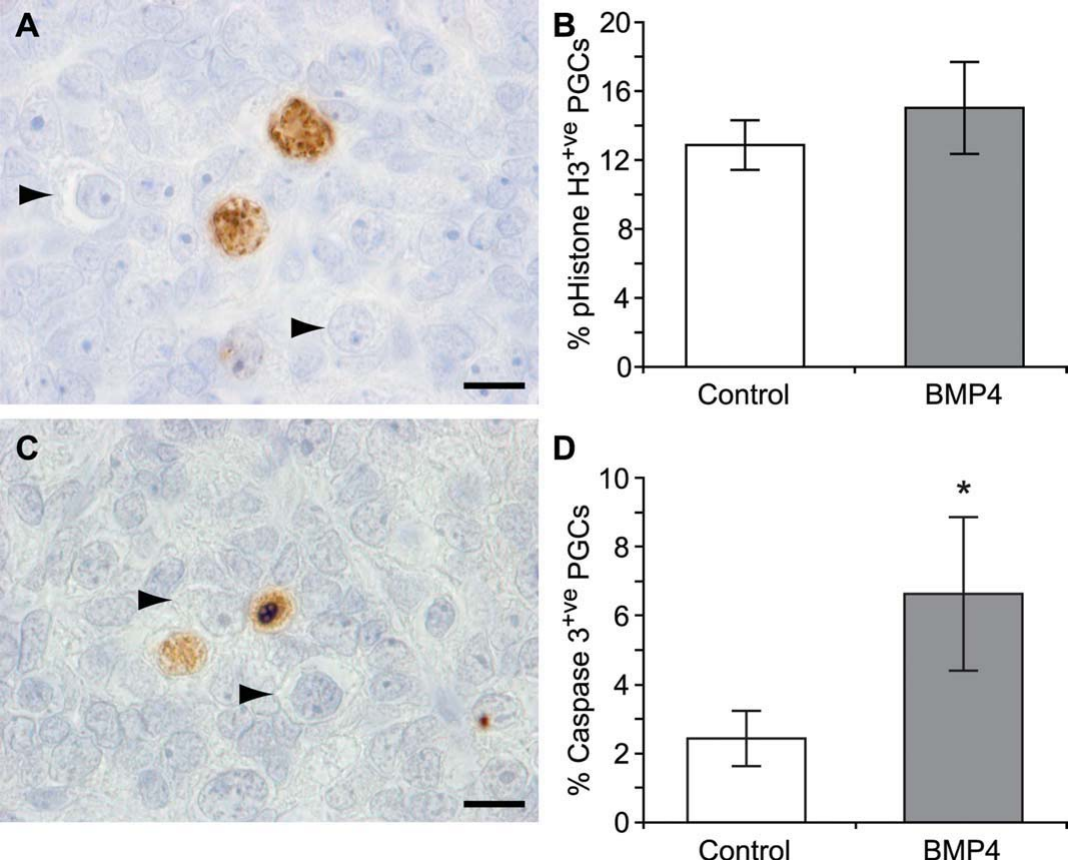
BMP4 promotes PGC apoptosis, but does not impair PGC proliferation. (**A**): Representative photomicrograph of a section of human fetal ovary cultured in the presence of BMP4 for 10 days and stained for proliferation marker phosphoHistoneH3 (brown staining). Nonproliferating germ cells are also detectable (arrowhead). (**B**): Stereological determination of phosphoH3-postitive PGC numbers revealed no difference in the proportion of PGCs staining positively for phosphoHistoneH3 between control and BMP4-treated groups, indicating no effect of BMP4 on PGC proliferation (*n* = 4, *p* > .05). (**C**): Representative photomicrograph of cultured human fetal ovary stained for the apoptosis marker cleaved caspase 3, showing immunopositive apoptotic PGCs (brown staining) and adjacent immunonegative PGCs (arrowhead). (**D**): The proportion of PGCs positively stained for cleaved caspase three was significantly increased in BMP4-treated cultures compared with controls (*n* = 4, *p* < .03), demonstrating a proapoptotic effect of BMP4 on PGCs in the human fetal ovary and indicating enhanced apoptosis, rather than impaired proliferation, is the cause of the reduced PGC numbers in BMP4-treated human fetal ovaries. Scale bars: (**A, C**) 10 μm. Abbreviations: BMP4, bone morphogenetic protein four; PGCs, primordial germ cells.

The reduction in PGC number and density in hrBMP4-treated gonads could arise from a decrease in PCG proliferation, increased PGC apoptosis, or a combination of both of these processes. We therefore performed immunohistochemistry and stereology using the proliferation marker phospho-H3 (Fig. 5A) on adjacent sections to those used to assess total germ cell numbers, to determine the number and proportion of germ cells undergoing proliferation. We detected no significant difference in the proportion of phospho-H3-positive PGCs between control and hrBMP4-treated ovaries (12.9% ± 1.4% in controls vs. 15.0% ± 2.7% in hrBMP4-treated ovaries, *n* = 4, *p* > .05; Fig. 5B), suggesting hrBMP4-treatment does not affect PGC proliferation.

We then repeated this approach to determine the proportion of apoptotic PGCs, by performing immunohistochemistry/stereology for cleaved caspase three (Fig. 5C). The proportion of caspase 3-positive PGCs was significantly elevated in hrBMP4-treated ovaries compared with untreated controls (6.6% ± 2.2% vs. 2.4% ± 2.2%, *n* = 4, *p* < .03; Fig. 5D), an approximately 2.8-fold increase. We therefore conclude that hrBMP4 negatively regulates PGC numbers in the human fetal ovary by promoting apoptosis rather than by inhibiting PGC proliferation.

### A BMP-Inducible Apoptosis-Related Gene Is Upregulated in BMP-Treated Human Fetal Ovaries

**Figure 6:**
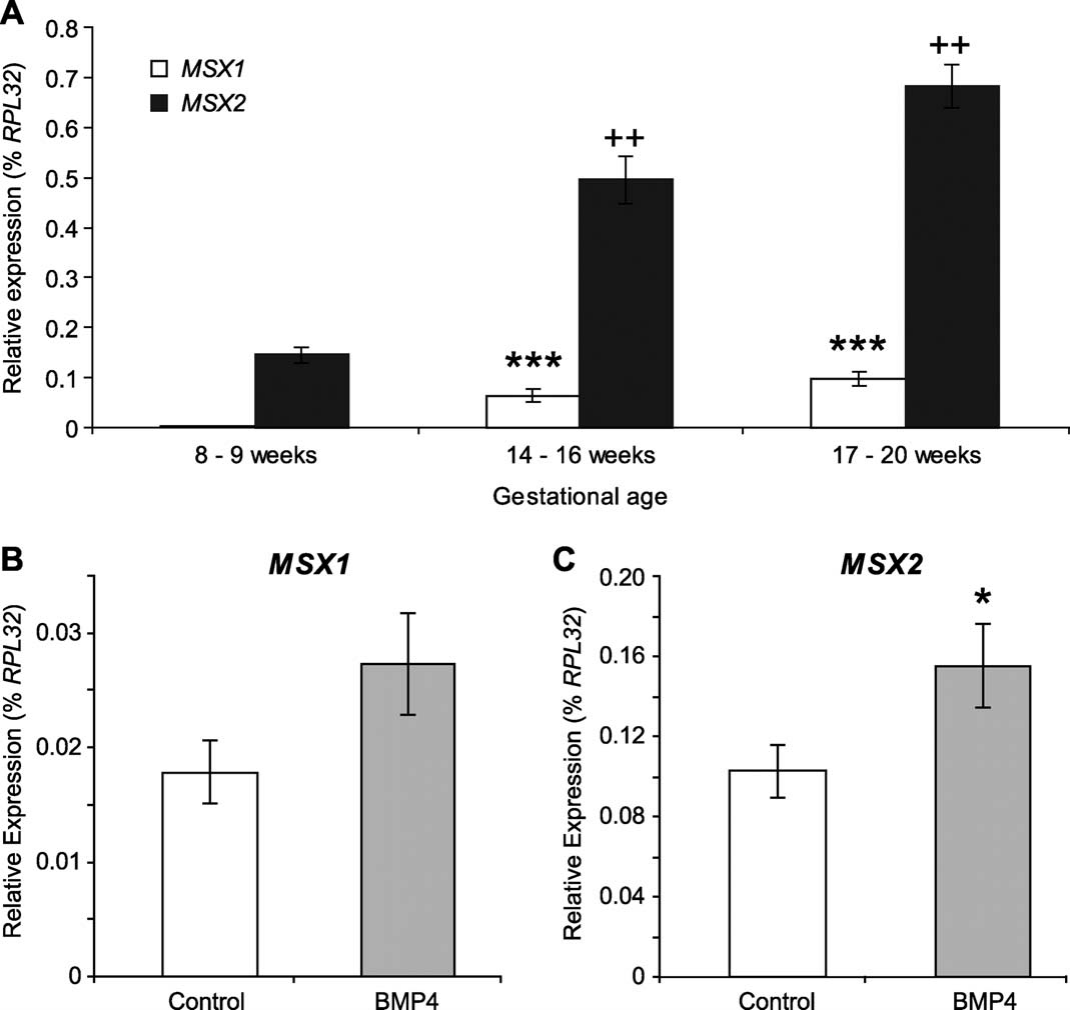
Developmentally regulated and BMP-inducible expression of *MSX* genes in the human fetal ovary. qRT-PCR analysis of *MSX1* and *MSX2* expression between 8 and 20 weeks gestation in the human fetal ovary reveals expression increases significantly with increasing gestation, from 8 to 9, 14 to 16, and 17 to 20 weeks gestation (**A**; *n* = 5–6 per group, ***p < .001, ^++^*p* < .01). Culture of human fetal ovary-mesonephros complexes with BMP4 for 24 hours induced a small but statistically insignificant increase in the expression of *MSX1* (**B**; *n* = 8, *p > .05). Expression of *MSX2*, associated with apoptosis in many developmental contexts, was significantly increased in BMP4-treated compared with controls (**C**; *n* = 8, *p < .05), however, suggesting a possible role of *MSX2* in BMP-induced PGC apoptosis in the human fetal ovary.

We first determined whether *MSX1* and *MSX2* were expressed in the human fetal ovary. Expression of both genes rose significantly over the gestational range examined (Fig. 6A). *MSX1* expression increased 44-fold between 8–9 and 14–16 weeks, (1.5 × 10^−3^ ± 4.8 × 10^−5^% vs. 6.4 × 10^−2^ ± 1.3 × 10^−2^% of *RPL32* expression, *p* < .001) concomitant with the onset of germ cell differentiation, and rose 66-fold over the entire gestational range examined. *MSX2* expression was substantially higher than that of *MSX1* at all gestations examined, and also increased significantly over the developmental window examined although the magnitude of the increase in expression was lower. *MSX2* expression increased sixfold over the gestational range examined (0.15% ± 0.02% at 8–9 weeks to 0.50% ± 0.05% at 14–16 weeks and 0.68 ± 0.04 at 17–20 weeks, *n* = 5–6, *p* < .01). Expression of both *MSX1* and *MSX2* therefore appears to be developmentally regulated in the human fetal ovary, with the expression of both genes substantially upregulated as germ cells make the transition from mitotic proliferation to meiotic differentiation.

We next examined whether *MSX* genes may be involved in the BMP-induced germ cell apoptosis detected in our cultures. We cultured human fetal ovary-mesonephros complexes in the presence or absence of BMP4, and examined the expression of *MSX1* and *MSX2* by qRT-PCR after 24 hours. BMP4 increased *MSX1* expression from 0.018% ± 0.003% to 0.027% ± 0.004% (*n* = 8; Fig. 6B), although this was not statistically significant. In contrast, the expression of *MSX2* was significantly increased in hrBMP4-treated ovaries compared with untreated controls (0.16 ± 0.02 vs. 0.10% ± 0.01%, *n* = 8, *p* = .05; Fig. 6C), indicating a possible role for *MSX2* in BMP-induced PGC apoptosis in the human fetal ovary.

## DISCUSSION

Somatic cell-derived growth factors are key regulators of germ cell fate at diverse developmental stages. The growth factor composition of the human fetal germline stem cell niche has not been extensively studied, however. In this article, we have identified the existence of a functional and developmentally regulated BMP signaling pathway in the human fetal ovary, identified germ cells to be the sole targets of BMP action in this organ, and revealed a proapoptotic role for BMP4 in regulating PGC development. Although previous studies have demonstrated that isolated human PGCs can be reprogrammed into pluripotent stem cells by the action of growth factors [50,51], we believe this to be the first report detailing the regulation of human PGC development by a growth factor in a physiologically relevant (i.e., gonadal niche) context.

We have observed developmentally regulated expression of the BMP ligands BMP2 and BMP4, with an increase in the expression of *BMP2* and a decrease in the expression of *BMP4* with increasing gestation (and increasing germ cell differentiation). It should be noted, however, that the change is not absolute, and by 17–20 weeks gestation, the levels of transcripts encoding BMP2 and BMP4 are comparable. It is therefore possible that BMP2 and BMP4 have distinct roles within the fetal ovary and further work will be needed to investigate whether their transcriptional targets, and their SMAD utilization, differ during ovarian development. In the mouse, *Bmp2* expression is restricted to the fetal ovary following sex determination and its expression driven by Wnt(4)-signaling [52]. The increasing expression of *BMP2* with ongoing ovarian differentiation reported here may reflect a similar mechanism in operation in the human fetal ovary. Mice deficient in Bmp7 have reduced numbers of germ cells due to a defect in PGC proliferation after colonization of the gonad, indicating Bmp7 to be an important component of the germ cell niche [13]. We found the expression of *BMP7* to be extremely low relative to that of *BMP2* and *BMP4*. Therefore, although we are unable to state definitively that BMP7 has no functional role in regulating human fetal germ cell development, the extremely low levels of transcript encoding this protein suggest this to be the case. Species-specific differences may therefore exist in the repertoire of BMPs expressed in the developing mammalian ovary.

BMPs form homo- and heterodimers that signal through heteromeric complexes of type I (BMPR1a, BMPR1b and ACTR1a, also known as ALK3, ALK6, and ALK2) and type II BMP receptors (BMPRII). Ligand binding triggers a phosphorylation cascade resulting in the activation of BMP-receptor regulated (BR-) SMADs 1, 5, and 8, enabling their association with SMAD4 and subsequent nuclear import [53]. Existing data on the expression of BMP receptors and BR-SMADs in the developing mammalian gonad is contradictory. Pesce et al. reported expression of transcripts encoding BmpR1a and Smad1 in both germ and somatic cells in the fetal mouse gonad, but were unable to detect Smad5 in purified PGCs [27]. Conversely, Pellegrini et al. demonstrated BmpR1a to be expressed exclusively by PGCs and found that only Smad5 localized to the nuclei of PGCs [46], indicating this BR-Smad to be the predominant transducer of BMP signals in germ cells at this stage. Our findings also demonstrate a broad expression of BMPR1a in the first trimester human fetal gonad, which becomes restricted to germ cells at later gestations, and (in contrast to an earlier publication [54]) germ cell-specific expression of BMPR1b all gestations examined. The pSMAD1/5 antibody used in these studies is unable to discriminate between the two proteins, but our finding that *SMAD5* expression is greatest in the first trimester around the time of PGC proliferation, and declines subsequently, may support the hypothesis that SMAD5 is the predominant transducer of BMP signals in undifferentiated early germ cells. It will also be of interest to determine whether the switch from BMP4 to BMP2 expression is related to the change in expression of SMAD1 and SMAD5. We have determined that BMP4 does not regulate *SMAD5* gene expression in the human fetal ovary (data not shown), but differential BR-SMAD utilization in response to different BMPs has been reported in other systems [55,56], including mouse spermatogonia [46]. Consistent with the data reported here, previous studies have also failed to detect expression of Smad8 in the fetal mouse gonad [27,46], suggesting the absence of a role for this factor in gonadal development is conserved.

A striking finding of this study is the relocalization of phosphorylated SMAD1/5 from the nucleus in PGCs to the cytoplasm in differentiating germ cells in syncitial clusters. This result reveals the existence of germ cell-intrinsic mechanisms that act negatively to regulate/attenuate BMP signaling by keeping phosphorylated BR-SMADs in the cytoplasm. This was not absolute as some germ cells showed both nuclear and cytoplasmic pSMAD1/5 staining. SMAD6 has been shown to negatively regulate BMP signaling by sequestering phosphorylated SMAD1 [47], but the restriction of SMAD6 expression to somatic cells of the fetal ovary rules this out as a possible mechanism. BMP signaling can be antagonized by the action of peptide growth factors, such as epidermal growth factor (EGF), which promotes the phosphorylation of the linker region of BR-SMADs by MAP kinases and inhibits their translocation to the nucleus [57]. It is unclear as to whether such a mechanism of attenuating BMP signaling could account for the near-complete relocalization of pSMAD1/5 from nucleus to cytoplasm in differentiating germ cells reported here. Importing β3, a member of a protein family implicated in the nuclear import of SMADs [58] relocalizes from the nucleus to cytoplasm as germ cells enter meiosis in the fetal mouse ovary [59], suggesting key changes may occur in the permeability of the nuclear membrane as germ cells differentiate. This may provide a possible explanation for the nuclear exclusion of pSMAD1/5 in oogonia, if similar changes occur as PGCs enter meiosis in the human fetal ovary. Interestingly, restricted SMAD nuclear import has recently been reported in Sertoli cells in response to activin, providing a mechanism by which the expression of subsets of downstream genes can be modulated selectively in response to growth factors with pleiotropic effects [60].

Targeted disruption of *Bmp4* in mice leads to a failure of germ lineage specification, thus the role of Bmp4 in regulating postmigratory PGCs cannot be studied in vivo. Previous studies have demonstrated that BMP4 can act as a mitogen for isolated mouse PGCs cultured on feeder cell layers in vitro [27], whereas culture of fetal mouse ovaries with BMP4 has been reported to induce a testicular-like phenotype and reduce meiotic cell numbers [26]. Our findings indicate that within the human fetal ovary, BMP4 negatively regulates PGC number by promoting apoptosis, suggesting that increased germ cell death may explain the reduction in the number of meiotic germ cells in BMP-treated fetal mouse ovaries reported by Ross et al. [26]. The most likely explanation for these differences is that PGCs display differential responses to growth factors when isolated and propagated on feeder layers, compared with when they are maintained within the gonad with the germ cell niche intact. Numerous examples of this have been described. Retinoic acid exerts potent mitogenic effects on isolated migratory and postmigratory mouse PGCs [61], but induces meiotic entry in germ cells cultured within intact fetal gonads obtained from mouse embryos at equivalent developmental stages [62,63]. Furthermore, signaling by IL-6 family cytokines, such as LIF, through the gp130 common cytokine receptor inhibits the entry into meiosis [18] and promotes the survival of isolated mouse PGCs in vitro [64], and inhibition of the receptor impairs PGC survival [17,19]. However, mice with homozyogous deletions of the gp130 receptor display only a mild, male-specific reduction in PGC number [20] and LIF-receptor deficient mice have a normal complement of PGCs [65]. Taken together with our finding that BMP4 promotes apoptosis of human PGCs rather than their proliferation or survival, these examples strongly suggest that maintaining isolated PGCs on feeders in vitro does not accurately recapitulate their normal gonadal microenvironment and responses in vivo, and supports the organ-culture approach as a method of for investigating growth factor signaling in the developing human gonad. The ability of our culture system to recapitulate the in vivo environment is further supported by the number and proportion of proliferating germ cells in our cultures, both of which are comparable to values determined empirically using age-matched human fetal ovaries [49]. It is important to note, however, that differences are likely to exist between our organ culture system and the in vivo situation; growth factors produced by neighboring tissues known to affect gonadal development may be absent or present at reduced levels in our culture system, and the oxygen concentration is likely to differ significantly (hypoxic in vivo compared with standard normoxic tissue culture conditions used in this study). The latter of these may have particular significance, as recent studies have demonstrated that stem cells often occupy hypoxic niches, and that O_2_ concentration exerts diverse effects on stem cell survival, self-renewal, and differentiation (reviewed in [66]).

BMP-induced apoptosis occurs in diverse developmental contexts and is frequently associated with the expression of *MSX2* [31,35,38,39]. The data presented here demonstrate both a proapoptotic role for BMP signaling in the regulation of postmigratory human PGCs and reveal this to be associated with increased *MSX2* expression. As MSX2 can promote exit from the cell cycle, it has been proposed that BMP-induced *MSX2* expression in proliferating undifferentiated cells may result in an intracellular conflict between proliferation and differentiation/quiescence that can only be resolved by apoptosis [39], and a similar conflict may occur within the undifferentiated, proliferating PGCs within our cultures when exposed to BMP4. Interestingly, the substantial increases in *MSX* gene expression with increasing gestation correlate with the onset of a major wave of germ cell apoptosis which occurs as syncitial nests of germ cells break down and primordial follicles begin to form. It is tempting to speculate that these factors may in some way be involved in regulating this process, either by promoting germ cell death, or regulating germ cell differentiation.

The data presented here have important implications for efforts to derive germ cells from human embryonic stem (hES) cells. BMPs have been shown to enhance the derivation of PGCs from human [67] and mouse [68] ES cells. At doses equivalent to those used in this study, BMP4 has been reported to increase the expression of pre- (*OCT4*, *NANOG*, *KIT*) and post- (*DAZL*, *VASA*) migratory PGC markers in differentiating ES cells [69]. Conversely, BMP4 treatment has also been reported to decrease *VASA* expression in human ES cells differentiated in long-term (21-day) monolayer cultures [70]. As VASA is expressed only by differentiating germ cells in the human fetal gonad [8], this finding suggests that BMP4 has a negative effect on the derivation of differentiated germ cells from hES cells, consistent with the loss of postmigratory PGCs in BMP-treated ovaries reported here. Our data suggest that sustained exposure to BMPs throughout the differentiation process could be detrimental to the survival of ES-cell derived germ cells and limit the efficiency of protocols to obtain more differentiated germ cell types.

## CONCLUSION

In summary, we have identified a functional and developmentally regulated BMP signaling system within the developing human fetal ovary and identified a proapoptotic role for BMP4 in the regulation of human postmigratory PGCs. The differences between these data and studies on isolated mouse PGCs underline the importance of studying germ cell development in multiple species and of utilizing physiologically representative systems. The data reported here extend our understanding of the growth factor composition of the human PGC niche and how germ cell numbers are regulated in the human fetal ovary, and will inform strategies for the differentiation of germ cells from pluripotent stem cells and improve culture systems for the differentiation of germ cells in vitro.
